# DNA mismatch repair gene MLH1 induces apoptosis in prostate cancer cells

**DOI:** 10.18632/oncotarget.2315

**Published:** 2014-08-06

**Authors:** Shinichiro Fukuhara, Inik Chang, Yozo Mitsui, Takeshi Chiyomaru, Soichiro Yamamura, Shahana Majid, Sharanjot Saini, Hiroshi Hirata, Guoren Deng, Ankurpreet Gill, Darryn K. Wong, Hiroaki Shiina, Norio Nonomura, Rajvir Dahiya, Yuichiro Tanaka

**Affiliations:** ^1^ Department of Surgery/Urology, Veterans Affairs Medical Center, San Francisco, California, United States of America; ^2^ Department of Urology, University of California, San Francisco, California, United States of America; ^3^ Department of Urology, Osaka University Graduate School of Medicine, Suita, Japan; ^4^ Department of Oral Biology,Yonsei University College of Dentistry, Seoul, Korea; ^5^ Department of Urology, Shimane University Faculty of Medicine, Izumo, Japan; ^6^ Department of Urology, Kagoshima University Graduate School of Medical and Dental Sciences, Kagoshima, Japan

**Keywords:** MLH1, c-Abl, apoptosis, prostate cancer

## Abstract

Mismatch repair (MMR) enzymes have been shown to be deficient in prostate cancer (PCa). MMR can influence the regulation of tumor development in various cancers but their role on PCa has not been investigated. The aim of the present study was to determine the functional effects of the mutL-homolog 1 (MLH1) gene on growth of PCa cells. The DU145 cell line has been established as MLH1-deficient and thus, this cell line was utilized to determine effects of MLH1 by gene expression. Lack of MLH1 protein expression was confirmed by Western blotting in DU145 cells whereas levels were high in normal PWR-1E and RWPE-1 prostatic cells. MLH1-expressing stable transfectant DU145 cells were then created to characterize the effects this MMR gene has on various growth properties. Expression of MLH1 resulted in decreased cell proliferation, migration and invasion properties. Lack of cell growth *in vivo* also indicated a tumor suppressive effect by MLH1. Interestingly, MLH1 caused an increase in apoptosis along with phosphorylated c-Abl, and treatment with MLH1 siRNAs countered this effect. Furthermore, inhibition of c-Abl with STI571 also abrogated the effect on apoptosis caused by MLH1. These results demonstrate MLH1 protects against PCa development by inducing c-Abl-mediated apoptosis.

## INTRODUCTION

Prostate cancer (PCa) is the most frequently diagnosed malignancy and the second leading cause of cancer death among males in the United States [1]. It is estimated that in 2014, there will be 233,000 new cases and 29,480 deaths due to PCa [1]. PCa is a disease of aging as 1 in 43 of those aged 50 to 59, 1 in 16 aged 60 to 69, and 1 in 9 aged 70 years and older will develop invasive disease [1]. Most cases of PCa are treatable with androgen-deprivation therapy however the majority of PCa recur as androgen-independent, metastatic disease that leads to death within several years. Currently, no effective therapies are available to cure androgen-independent PCa. Thus, new prognostic markers and effective treatment strategies are urgently needed.

A factor that may prevent the prostate carcinogenesis process is the DNA mismatch repair (MMR) system that consists of various types of proteins such as the MutS homologues and MutL homologues (MLH) [2, 3]. Defects in this MMR pathway result in an increased rate of mutation or genetic instability, which in turn leads to defects in genes that regulate cell proliferation and death [4], thereby increasing susceptibility to cancers. Alternatively, MMR genes have also been shown to sensitize cells toward DNA lesions and trigger apoptosis, cell cycle arrest, and cell death [5, 6]. Thus, MMR is essential for proper cellular function and health of the individual.

Clinical case reports have shown that PCa can occur in men afflicted with hereditary non-polyposis colorectal cancer (HNPCC), a disease known for mutations in MMR [7, 8]. The cumulative lifetime risk of PCa was calculated to be two-fold higher among individuals with HNPCC and defective MMR as compared to the general population [8]. In a study by Grindedal et al [9], nine individuals contracted PCa among a cohort of 106 Norwegian men described as carriers or obligate carriers of MMR mutation and this rate was much higher than the 1.52 expected to occur by chance in the Norwegian population (*P* < 0.01). These men were younger at the time of diagnosis (60.4 years vs. 66.6 years, *P* = 0.006) and had higher Gleason scores of 8 to 10 compared to men diagnosed prior to 70 years of age in the population (*P* < 0.00001). Kaplan-Meier analysis revealed that the cumulative risk of PCa diagnosis by age 70 years was 30% in MMR gene mutation carriers compared to 8% in the population. Thus MMR imperfections can lead to PCa.

Among the various MMR genes, hundreds of mutations and polymorphisms have been identified and interestingly, most of these variants are observed in the MLH1 gene (50%), thus making this a highly susceptible gene in the carcinogenesis process [10]. Defects in MLH1 have been documented in various cultured prostate cell lines. Among the first reports was the PCa line DU145 where Boyer et al [11] identified a mutation in the MLH1 gene after demonstrating reduced MMR activity and microsatellite instability. Chen et al [12] confirms genomic instability in DU145 cells where MLH1 protein expression was lacking. A prior study from our laboratory [13] show the DU145, LNCaP, and PC3 PCa cell lines to have much lower levels of MLH1 protein expression and these all had reduced DNA repair activity as compared to the MMR proficient Hela cells.

MLH1 expression has also been studied in prostate tissue. A report by Chen et al [12] found that in the normal prostate gland, MLH1 protein was predominantly detected in the nuclei of glandular luminal epithelium, basal cells, and some stromal cells. This pattern of MLH1 expression was also observed in the normal adjacent region of prostate tumor tissue. In malignant prostate cells however, MLH1 levels were found to be less than that observed in normal adjacent areas. Other studies also show reduced MLH1 protein expression in prostate tumor regions as compared to normal adjacent [14, 15]. Additionally, MLH1 expression was found to be much lower in PCa when compared to benign prostatic hyperplasia tissue [15].

The MLH1 gene is thus shown to be reduced in PCa cell lines and tissues. Therefore in this report, we characterize the functional role the MLH1 gene plays in PCa cells. Our results are the first to show that re-expressing the MLH1 gene in PCa cells causes inhibition of cell growth both *in vitro* and *in vivo*. Also, we demonstrate MLH1 to enhance apoptosis and induce phosphorylation of the c-Abl protein as a mechanism for its protective role in PCa cells.

## RESULTS

### Prostate cancer cell line DU145 is MLH1 deficient

**Figure 1:**
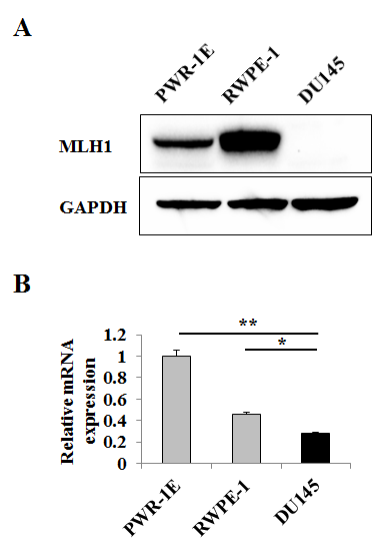
MLH1 expression is downregulated in DU145 PCa cells Prostatic cell lines were grown in culture dishes for two days. (A) Representative immunoblot displaying MLH1 protein expression in DU145 and normal prostate epithelial PWR-1E and RWPE-1 cells. GAPDH was used as loading control. (B) Relative mRNA expression levels of MLH1 in cell lines as determined by real-time PCR. Expression levels are normalized to PWR-1E. Data are presented as mean±SEM of three experiments. Asterisks denote differences between the compared values: *P<0.05, **P<0.01.

Prior studies have established DU145 cells to be MLH1-deficient [12, 13] and thus, we selected this cancerous cell line for further studies. Initially, we confirmed protein and RNA expression levels of MLH1 in DU145 cells and as a comparison, measured expression in the normal prostate epithelial cell lines, PWR-1E and RWPE-1. Figure 1A shows no protein detected and 1B significantly reduced RNA expression for MLH1 in DU145 cells whereas levels were high in both PWR-1E (P<0.01) and RWPE-1 (P<0.05).

### Effect of MLH1 re-expression on cell proliferation, migration and invasion

**Figure 2:**
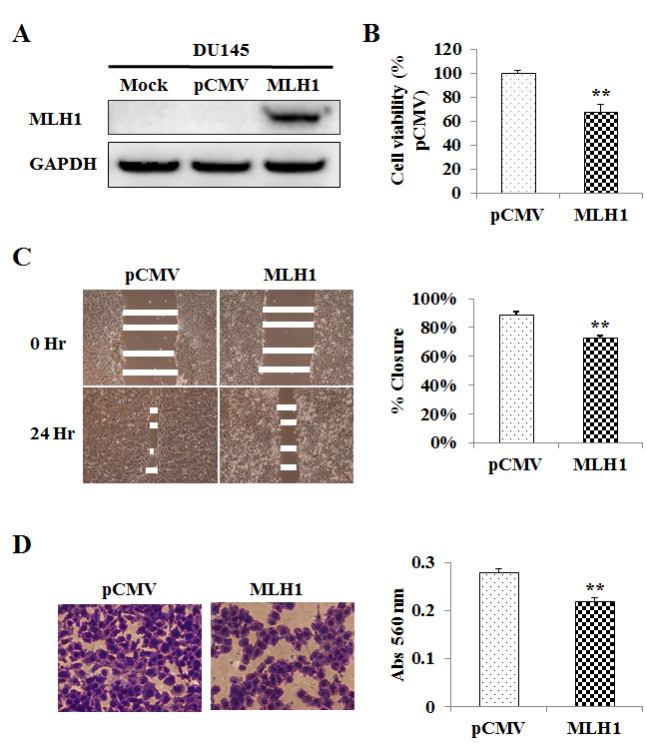
Re-expression and tumor suppressive effect of MLH1 on DU145 cells (A) Ectopic expression of MLH1. DU145 cells stably transfected with either MLH1 or empty vector (pCMV) along with mock (parental DU145 cells treated with transfection reagent alone) were grown for 48 hours and underwent Western analyses. GAPDH was used as loading control. (B) Cell proliferation as analyzed by the MTS cell proliferation assay 48 hours after plating cells. Results are expressed as % and normalized to pCMV control. (C) Cell migration as measured by wound healing assay. A wound was formed by scraping culture dishes using a pipet tip and closure measured after 24 hours. *Left:* Representative images of wound healing assay are shown. *Right:* Migration expressed as % closure of wound. (D) Cell invasiveness as measured using Matrigel. Cells were placed onto transwell membrane and allowed to invade for 24 hours. *Left:* Representative images of invading cells are shown. *Right:* Cell invasiveness as measured by absorbance (Abs) at 560 nm. Data are presented as mean±SEM of at least three experiments; **P<0.01 MLH1 versus pCMV.

Absence of MLH1 protein in DU145 cells led us to examine whether re-expressing MLH1 affects the growth of these cells. To do this, we established a DU145 cell line that stably expresses MLH1. As shown in Figure 2A, MLH1 protein was detected by Western blotting in stable MLH1-transfected DU145 cells but not in mock or vector control cells. To determine the effects of MLH1 on cellular properties, we conducted various functional analyses. MTS assay show that cell proliferation was reduced over 30% after 48 hours in DU145 cells expressing MLH1 compared to vector control (P<0.01) (Figure 2B). Wound healing assay demonstrated significant inhibition of cell migration in MLH1-stable compared with vector-transfected DU145 cells after 24 hours (73% versus 89% closure, respectively, P<0.01) (Figure 2C). Matrigel invasion assay also show that the number of invading cells was significantly decreased in MLH1 transfectants with absorbance at 560 nm of 0.22 compared with vector control having absorbance of 0.28 (P<0.01) (Figure 2D). These results indicate MLH1 to play an important role in reducing tumor cell proliferation, migration and invasion.

### Effect of MLH1 expression on tumorigenicity *in vivo*

**Figure 3:**
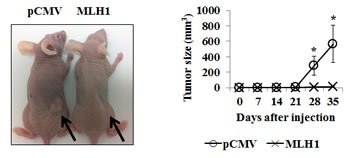
Expression of MLH1 suppresses growth of DU145 cells *in vivo* Athymic nude mice were injected subcutaneously with stable MLH1 or pCMV-transfected DU145 cells and growth monitored over time. *Left:* Representative image of tumors in mice five weeks after injection of cells. *Right:* Growth of tumor size plotted over time. Data are presented as mean±SEM of five mice per group; *P<0.05 MLH1 versus pCMV for each time point.

To validate the suppressive effect of MLH1 under *in vitro* conditions, we also determined effects of MLH1 on tumor growth in animal models. Stable MLH1 and pCMV DU145 cells were subcutaneously injected into nude mice. We observed that expression of MLH1 inhibited DU145 cell tumor formation throughout the duration which lasted 5 weeks whereas by week 4, tumor growth was visible in pCMV animals. By week five, tumor sizes were dramatically increased to an average of 565 mm^3^ in controls compared to 13 mm^3^ in MLH1-treated mice (P<0.05) (Figure 3). These results suggest MLH1 suppresses PCa cell growth *in vivo*.

### MLH1 influences cellular apoptosis

**Figure 4:**
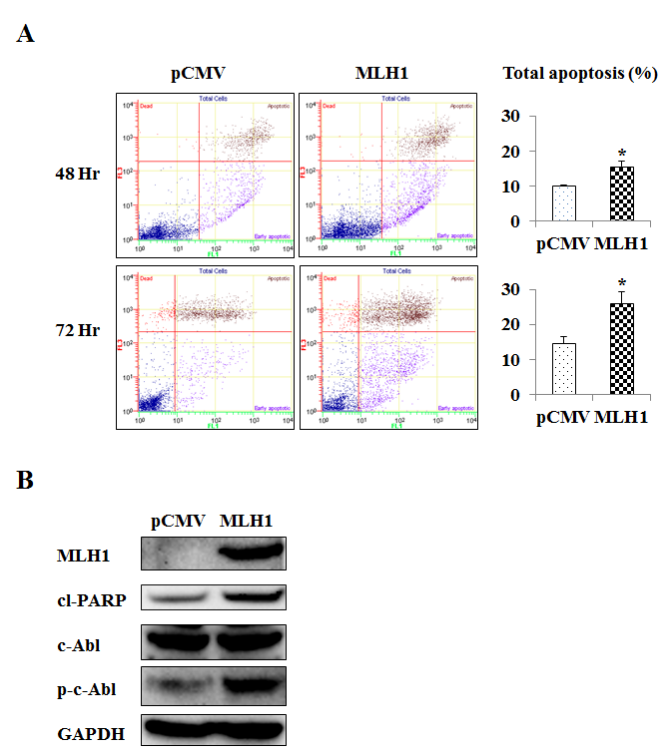
MLH1 expression upregulates apoptosis and phosphorylated c-Abl (p-c-Abl) Stable MLH1-expressing and vector control DU145 cells were grown over time. (A) Apoptosis as measured by flow cytometric analyses. Representative biparametric histogram showing cell population in early (bottom right quadrant) and late (top right quadrant) apoptotic, and viable (bottom left quadrant) states. *Left:* pCMV control, *Middle:* MLH1-expressing, *Right:* Total apoptosis %, *Upper:* 48 hours of growth, *Lower:* 72 hours of growth. Bar graph of total apoptosis % is presented as mean±SEM of three experiments, *P<0.05 MLH1 versus pCMV. (B) Protein levels of MLH1, cleaved PARP (cl-PARP), c-Abl, and p-c-Abl were determined by Western blot analyses after growing pCMV and MLH1 cells for 48 hours. GAPDH was used as loading control.

Since MLH1 restoration significantly inhibits cell growth and progression of DU145 cells both *in vitro* and *in vivo*, we hypothesized that its expression may induce apoptosis. Figure 4A shows that the apoptotic and early apoptotic fractions (upper right and lower right quadrants of biparametric histograms, respectively) were increased in MLH1-transfectants compared to vector control after 48 and 72 hours of growth. Total apoptosis levels were 10.3% versus 15.7% at 48 hours (P<0.05) and 14.6% versus 26.0% at 72 hours (P<0.05) for pCMV versus MLH1-expressing cells, respectively (Figure 4A).

### MLH1 induces c-Abl phosphorylation

The pro-apoptotic role for MLH1 observed suggests that apoptotic pathways are affected. It has been reported that c-Abl mediates MLH1-dependent apoptosis in other cell types such as colon cancer cells [16]. Therefore, we examined the expression of c-Abl in DU145 cells and found that phosphorylated c-Abl protein was up-regulated in MLH1-transfectants whereas levels of the non-phosphorylated form was not changed (Figure 4B). Additionally, we found that MLH1 expression caused an increase in cleaved PARP which further supports the pro-apoptotic role of MLH1. These results indicate c-Abl phosphorylation to moderate the apoptotic effects of MLH1 in PCa cells.

### Inhibition of MLH1 prevents apoptotic cell death in MLH1-expressing cells

**Figure 5:**
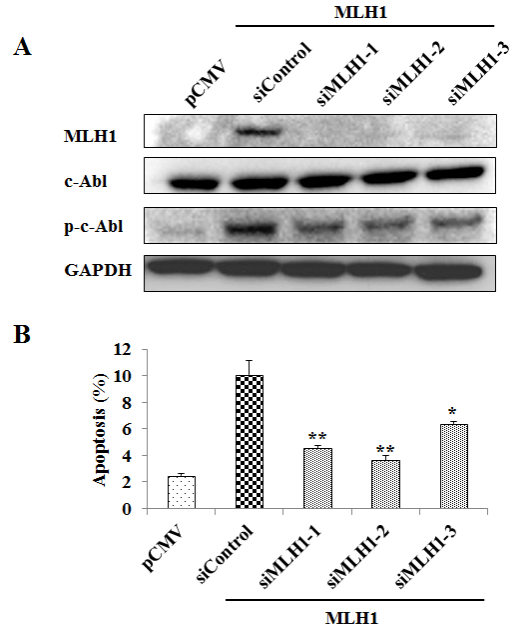
MLH1 knockdown reduces phosphorylated c-Abl (p-c-Abl) and apoptosis Three MLH1 siRNAs (siMLH1s) were transfected individually along with a non-specific siRNA control (siControl) into stable MLH1-expressing DU145 cells for 48 hours. pCMV control cells treated with Lipofectamine are also included. (A) Protein levels of MLH1, c-Abl, and p-c-Abl in cells as determined by Western blot analyses. GAPDH was used as loading control. (B) Total apoptotic cells were analyzed by flow cytometry. Data are expressed as mean±SEM of three experiments; *P<0.05 **P<0.01 siMLH1s versus siControl.

To establish MLH1 as a key effector in the c-Abl-mediated apoptotic signaling pathway, we measured apoptotic cell death by exploiting siRNA to selectively knock down MLH1 (siMLH1) in MLH1-stable DU145 cells. We first confirmed the ability of siMLH1 to suppress endogenous MLH1 levels. Administration of three MLH1 siRNAs to MLH1-expressing DU145 cells decreased both basal MLH1 mRNA (data not shown) and protein (Figure 5A) expression. Interestingly, all three siMLH1s also significantly reduced apoptosis (siMLH1-1 (4.6%, P<0.01), siMLH1-2 (3.6%, P<0.01), siMLH1-3 (6.4%, P<0.05) compared to siRNA negative control (10.1%) (Figure 5B). Furthermore, MLH1 knockdown decreased phosphorylation of c-Abl without affecting non-phosphorylated c-Abl protein levels (Figure 5A). These results demonstrate that MLH1 induces apoptosis by c-Abl phosphorylation in PCa cells.

### Inhibition of c-Abl prevents apoptotic cell death in MLH1-expressing cells

**Figure 6:**
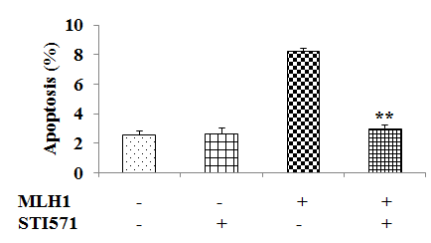
Inhibition of c-Abl nullifies MLH1-triggered apoptotic effect MLH1-expressing and pCMV control DU145 transfectants were treated with either DMSO or STI571 and maintained for 48 hours. Total apoptotic cells were then analyzed by flow cytometry. Data are expressed as mean±SEM of three experiments; **P<0.01 MLH1+/STI571+ versus MLH1+/STI571-.

To confirm the role of c-Abl as a regulator of MLH1-stimulated apoptosis, we examined the effect of the c-Abl inhibitor, STI571. MLH1 causes apoptosis in DU145 cells but when treated with STI571, a marked decrease of apoptosis to vector control levels was observed in MLH1-expressing cells (P<0.01) (Figure 6). We also observe phosphorylated c-Abl to be reduced due to STI571 (data not shown). These results further demonstrate that c-Abl regulates apoptosis in MLH1-expressing DU145 PCa cells.

## DISCUSSION

The MMR system recognizes and corrects mis-incorporated nucleotides and insertion/deletion mis-pairs formed during DNA replication [2, 3]. Loss of proteins critical in this repair process however, can result in genetic alterations that may ultimately lead to cancer. MMR deficiency also predisposes cells of the body to an increased frequency of inactivating mutations in genes important for suppressing carcinogenesis [4]. Besides their established role in DNA repair, MMR genes are also involved in cell cycle checkpoints and apoptosis stimulated by DNA damage [5, 6]. Resistance to apoptosis and cell death were shown in cells deficient in MMR genes when treated with DNA damaging chemotherapeutic agents [17, 18]. The MMR system is thus vital for maintaining cell integrity and disease prevention.

Among the various MMR genes, MLH1 has been shown to be among the most essential in sensitizing cells to undergo apoptosis [19]. This gene has also been shown to be more prone to mutation [10]. The genetic status of MLH1 in cells thus has high impact in cellular function. The MLH1 gene is located in chromosome 3p21 region, consists of 19 exons and is roughly 58 kilobases in length [20]. MLH1 is a nuclear protein that consists of 756 amino acids with a size of 80 kDa.

In prostate tissues, MLH1 has been shown to be downregulated in tumor regions as compared to normal prostate [12], normal adjacent regions [12, 14, 15] and benign hyperplasia [15]. Given that MLH1 can affect cell growth parameters, we explored the functional significance the MLH1 gene has on PCa cells which has never been done. MLH1 protein expression in the DU145 cell line has previously been shown to be downregulated [12, 13] and our results confirm this lack of protein expression. This reduction is apparently due to a truncation in the MLH1 gene caused by a premature stop codon as observed by Chen et al [12]. These investigators discovered a loss of 5 nucleotides at the start of the coding region of exon 2 that results in a frameshift producing a TGA stop at codon 39. Additionally, we observed that RNA levels are reduced in this cell line which indicates that the transcriptional machinery in DU145 cells is also affected. Thus, DU145 PCa cells are an ideal cell line to determine functional effects of the MLH1 gene by re-expression.

By expressing the MLH1 gene in DU145 cells, we observed that this gene caused a significant reduction in proliferation, migration, and invasive properties of this PCa cell line. A similar effect was observed *in vivo* as MLH1-expressing cells inhibited cell growth. This repressive activity was found to be due to apoptosis as MLH1 cells had a much higher rate compared to that of vector control. The apoptotic environment was also supported by an observed rise in cleaved PARP, suggesting the presence of caspases [21, 22]. On the contrary, silencing MLH1 in normal prostatic PWR-1E cells caused an increase in proliferation for two of three siMLH1 treatments (Supplementary Figure 1). Thus, MLH1 plays a tumor suppressor role in PCa cells. In concordance with our results, MLH1 has been shown by others to play an important role in apoptosis and cell cycle control [5, 6, 23]. In MLH1-deficient HCT116 colon cancer cells, expressing MLH1 by injecting chromosome 3 led to growth arrest whereas parental HCT116 or HCT116 cells injected with chromosome 2 had no effect after treatment with methylnitronitrosoguanidine (MNNG) [24]. Hawn et al [25] finds these HCT116+chromosome 3 cells to be arrested at G2 phase after treatment with 6-thioguanine or MNNG. In the ovarian cancer cell line A2780MNU1 that is MMR deficient, re-expression of MLH1 by gene transfection resulted in marked reductions of cell survival and proliferation, with higher percentage of cells displaying active caspase 3 after drug treatment [26]. In a report by Zhang et al [19], MLH1 cDNA was expressed in HCT116 and they find apoptosis to be increased nearly 3-fold. Also, Ruzov et al [27] observes that expressing MLH1 causes apoptosis in HCT116 cells as well as during the onset of gastrulation in embryos.

The mechanism by which MLH1 induces apoptosis thereby protecting against tumor formation is not fully understood. There are several studies that establish the c-Abl protein as a mediator in the MLH1-dependent cellular response to damage [16, 28-30]. Therefore in this study, we investigated whether c-Abl plays a crucial role in the MLH1-induced apoptotic response in PCa cells. We demonstrate phosphorylation of c-Abl to be a key component in MLH1-triggered apoptosis in DU145 cells as knockdown of c-Abl with the inhibitor STI571 abrogated the apoptotic effect in these cells. Also, the phosphorylated form of c-Abl is necessary as no differences were detected with the non-phosphorylated form between treatment groups. It is noteworthy to point out however, that although siMLH1 efficiently reduced MLH1 expression to pCMV levels, phosphorylated c-Abl was reduced but not to the same effect. This may be due to the fact that siMLH1 treatment started during the growth phase and cells had already accumulated phosphorylated c-Abl to an extent. This is further corroborated by the observation that apoptosis was also dramatically reduced but not to that of pCMV levels by all three siMLH1 treatments. The phosphorylation of c-Abl is thus critical for its activation and has been shown to occur at tyrosine sites [31, 32]. Further, our antibody detects phosphorylation at tyrosine-204 of the SH2 domain and studies show phosphorylation of this core region of c-Abl to impact protein conformation and signaling [33]. The process by which c-Abl is phosphorylated is not known. Studies have shown that activation of c-Abl is dependent on the ataxia telangiectasia mutated (ATM) gene [34, 35]. In a study by Baskaran et al [35], ATM was required to activate c-Abl and was due to phosphorylation at serine-465. Interestingly, a feedback for activation between these molecules was also observed [36].

The dependence of c-Abl in the MLH1 signaling pathway is in agreement with others as Li et al [16] observed MLH1-expressing cells to cause apoptosis in a p53-independent manner but when treating cells with STI571 or creating stable c-Abl knockdown transfectants, MLH1-dependent apoptosis was prevented. These authors further show the MLH1/c-Abl downstream effectors p73α and GADD45α to be of importance in the apoptotic pathway. In a report by Kim et al [29], MLH1 was also found to trigger the MEKK-1/MKK4/JNK/c-jun pathway in response to DNA damage. Interestingly, they find that c-Abl is required to activate this signaling cascade by MLH1. In fact, they observe a functional interacting complex between MLH1 and c-Abl. Thus although it is apparent that MLH1 affects alternative signaling pathways, evidence strongly suggests that c-Abl is a critical mediator for the MLH1-induced response.

In summary, this is the first report that shows MLH1 to display a protection against PCa by inhibiting cell proliferation, migration, and invasion *in vitro* as well as tumor growth *in vivo*. MLH1 induced apoptosis and was dependent on phosphorylation of c-Abl. Thus, we conclude that phosphorylated c-Abl plays a key role in the tumor suppressive effect of the MLH1 signaling pathway in prostate cells.

## MATERIALS AND METHODS

### Cell lines and reagents

Human PCa cell line DU145 and non-malignant epithelial prostate cell lines PWR-1E and RWPE-1 were purchased from American Type Culture Collection (Manassas, VA). Keratinocyte serum-free medium, bovine pituitary extract and human recombinant epidermal growth factor were purchased from Invitrogen (Carlsbad, CA). RPMI 1640, Opti-minimum essential medium and penicillin/streptomycin were obtained from the UCSF Cell Culture Facility (San Francisco, CA). Fetal bovine serum (FBS) was a product of Atlanta Biologicals (Lawrenceville, GA).

### Cell culture

DU145 cells were cultured in RPMI 1640 medium supplemented with 10% FBS. PWR-1E and RWPE-1 cells were cultured in keratinocyte growth medium supplemented with 5 ng/mL human recombinant epidermal growth factor and 0.05 mg/mL bovine pituitary extract. All cell lines were maintained at 37°C in a humidified atmosphere composed of 5% CO_2_ and 95% air.

### Western blot analysis

Whole cell extracts from cultured cells were prepared using radioimmunoprecipitation assay buffer (Thermo Scientific, Rockford, IL) containing protease inhibitor cocktail (Roche Diagnostics, Basel, Switzerland). Protein quantification was done using a BCA protein assay kit (Thermo Scientific) according to the manufacturer's instructions. Total protein (20 μg) was loaded onto 4-12% bis–tris gels with 3-(*N*-morpholino) propanesulfonic acid buffer and separated by a NuPAGE electrophoresis system (Invitrogen). Protein was transferred to Invitrogen™ polyvinylidene difluoride and immunoblotting was carried out according to standard protocols. Monoclonal antibodies against MLH1, cleaved PARP, c-Abl, and phosphorylated c-Abl were purchased from Cell Signaling (Danvers, MA) and monoclonal antibody against GAPDH (Santa Cruz Biotechnology, Santa Cruz, CA) was used to confirm equal loading. The membrane was washed and then incubated with secondary antibodies conjugated to horseradish peroxidase (Cell Signaling Technology). Protein complexes were visualized with the Echo-chemiluminescence (ECL) Detection System (GE Healthcare, Little Chalfont, UK) using the Chemidoc Imaging System (Bio-Rad Laboratories, Hercules, CA).

### RNA extraction

Total RNA was extracted from DU145, PWR-1E and RWPE-1 cells using the RNeasy Mini kit (Qiagen, Valencia, CA) according to the manufacturer's instructions.

### Quantitative reverse transcription–polymerase chain reaction

Extracted RNA was reverse-transcribed into complementary DNA (cDNA) using iScript cDNA Synthesis kit (Bio-Rad) and TaqMan MicroRNA Reverse Transcription kit (Applied Biosystems, Foster City, CA). Quantitative real-time PCR analysis was performed with an Applied Biosystems Prism 7500 Fast Sequence Detection System using TaqMan Universal PCR master mix according to the manufacturer's protocol (Applied Biosystems). Levels of RNA expression were determined using the 7500 Fast System SDS software version 1.3.1 (Applied Biosystems). PCR parameters for cycling were as follows: 95°C for 20 seconds, then 40 cycles of 95°C for 3 seconds and 60°C for 30 seconds. All reactions were done in a 10 μL reaction volume in triplicate. The data were analyzed using the delta-delta Ct method to calculate the fold-change. TaqMan probes and primers for MLH1 (assay ID: Hs00179866_m1) and GAPDH (assay ID: Hs02758991_g1) were obtained from Applied Biosystems. GAPDH was used as internal control.

### Establishment of DU145 cells stably expressing MLH1

DU145 cells were transfected with pCMV6-ENTRY vector expressing the C-terminally Myc and Flag-tagged human MLH1 cDNA as well as empty pCMV6-ENTRY vector as a control (OriGene Techologies, Rockville, MD) using X-treme Gene HD transfection reagent (Roche Diagnostics, Indianapolis, IN) according to the manufacturer's protocol. Clones were selected using 500 μg/ml of G418 (Invitrogen). Colonies resistant to G418 appeared within 2 weeks and single colonies were picked and then expanded for another 3 weeks to make stable clone stock cells.

### Cell proliferation assay

An MTS based assay was utilized to determine cell proliferation. Cells were plated in triplicate in 96-well microplates at a density of 5 × 10^3^ cells per well. At the desired time point, the number of viable cells was determined by adding CellTiter 96 AQueous One Solution reagent (Promega, Madison, WI) to each well and measuring the absorbance at 490 nm on a SpectraMax 190 plate reader (Molecular Devices, Sunnyvale, CA). Results were expressed as the percentage of optical density with absorbance of control cells being 100%.

### Migration and Invasion assays

Cell migration was evaluated by a wound-healing assay. Cells were plated in six-well dishes and monolayers were scraped using a P-20 micropipette tip. The width of the initial gap (0 hour) and the residual gap 24 hours after wounding were calculated from photomicrographs taken using a Nikon Eclipse TS100 microscope (Technical Instruments, Burlingame CA). Cell invasion properties were measured by a procedure using the CytoSelect Invasion assay (Cell Biolabs, San Diego CA) along with modified Boyden chambers consisting of transwell-precoated Matrigel membrane filter inserts with eight micron pores in 24-well tissue culture plates (BD Biosciences, Bedford, MA). Minimum essential medium containing 10% FBS in the lower chamber served as the chemo-attractant. After 24 hours, invasive cells were stained and observed by photomicrographs taken with the Nikon microscope and quantified using the SpectraMax plate reader at absorbance of 560 nm.

### *In vivo* tumor growth

All animal care was in accordance with the guidelines and this study was approved by the San Francisco Veterans Affairs IACUC (Institutional Animal Care and Use Committee). Animal users completed training programs to handle and work with mice through AALAS (American Association for Laboratory Animal Science) prior to animal experiments. For the subcutaneous xenograft mouse model, DU145 cells (5 × 10^6^) stably transfected with MLH1 or empty pCMV6-ENTRY vector were suspended in 100 μL RPMI 1640 medium and subcutaneously injected into the right backside flank of five week old female nude mice (strain BALB/c nu/nu; Charles River Laboratories, Wilmington, MA). Five nude mice were used per treatment group and tumor growth was examined over the course of 35 days. Tumor volume was calculated on the basis of width (x) and length (y) using the formula: x^2^y/2, where x<y.

### Apoptosis assay

Apoptosis was analyzed with an annexin V-fluorescein isothiocyanate (FITC)/7-amino-actinomycin D staining system obtained from BD Biosciences (San Diego, CA). Briefly, prostate cells were harvested and resuspended in binding buffer at a concentration of 1 × 10^6^ cells/ml. For each assay, 1 × 10^5^ cells were incubated with 5 μl of annexin V-FITC and 5 μl of 7-amino-actinomycin D in the dark for 15 min at room temperature. After adding 400 μl of binding buffer, samples were analyzed within an hour by a Cell Lab Quanta™ SC MPL flow cytometer (Beckman Coulter, Fullerton, CA).

### Small interfering RNA transfection

Cells grown to 30–50% confluence were treated with three different sets of small interfering RNA (siRNA) duplexes specific for human MLH1 (siMLH1) or universal scrambled negative control (OriGene Techologies) by transfection using Lipofectamine 2000 transfection reagent (Invitrogen) as described by the manufacturer. Knockdown efficiency was evaluated by both real-time PCR and Western blotting for the three siMLH1s at different concentrations and the optimal condition selected was 30 nM. Vector control cells treated with Lipofectamine alone was also included.

### STI571 treatment

pCMV and MLH1-transfected DU145 cells were grown in six-well plates. After two days, cells were treated with either 10 μM STI571 (via Fisher Scientific, Pittsburg PA) or dimethylsulfoxide (DMSO) diluent (Sigma-Aldrich, St Louis MO). Cells were then allowed to grow for another two days and harvested to determine apoptosis as described above.

### Statistical analysis

Values in are presented as the mean±standard error of mean (SEM) based on results obtained from at least three independent experiments. For *in vivo* studies, results are based on five animals per group. The relationship between variables was analyzed using the non-parametric Mann-Whitney U test or two-tailed Student's t-test. All analyses were performed using Expert StatView (version 4, SAS Institute, Cary, NC).

## SUPPLEMENTARY MATERIAL FIGURE


