# Supplementary material for: DNA mismatch repair gene MLH1 induces apoptosis in prostate cancer cells
Source: Oncotarget. 2014 Aug 6;5(22):11297–307. doi: 10.18632/oncotarget.2315 (PMC4294331; doi:10.18632/oncotarget.2315)
Supplement: Supplementary file 1 [file oncotarget-05-11297-s001.pdf]

DNA mismatch repair gene MLH1 induces apoptosis in prostate cancer cells

Supplementary Material

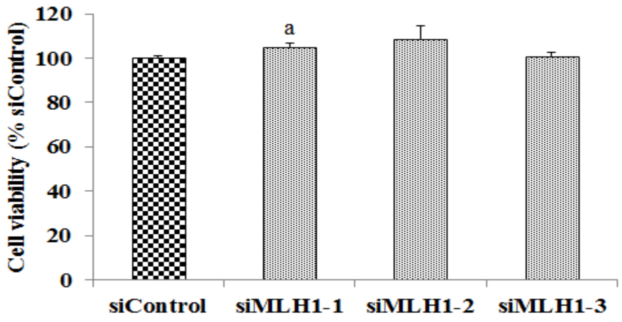

Supplementary Figure 1: Effect of MLH1 knockdown on proliferation of normal prostatic cells. Three MLH1 siRNAs (siMLH1s) were transfected individually along with a non-specific siRNA control (siControl) into PWR-1E cells for 48 hours and proliferation was analyzed by the MTS cell proliferation assay. Data are presented as mean±SEM of three experiments and expressed as % and normalized to siControl; [a]P=0.08 siMLH1-1 versus siControl.
